# Association of periprocedural phentolamine infusion with favorable outcome in patients with chronic kidney disease and chronic coronary syndrome undergoing coronary catheterization: a prospective randomized controlled pilot study

**DOI:** 10.1186/s12882-022-03050-9

**Published:** 2022-12-31

**Authors:** Mohamed abo Hamila, Helmy El Ghawaby, Mohamed Zaki, Mohamed Soliman, Khaled Gabr

**Affiliations:** 1 Critical Care Medicine, Beni Suef University, Beni Suef, Egypt; 2 Critical Care Medicine, Cairo University, Cairo, Egypt; 3 Cardiovascular Medicine, National Heart Institute, Giza, Egypt

**Keywords:** Contrast induced acute kidney injury, Phentolamine, Coronary catheterization

## Abstract

**Background:**

Chronic kidney disease (CKD) is a major risk factor for contrast induced acute kidney injury (CI-AKI) in chronic coronary syndrome (CCS) patients undergoing coronary catheterization. We aimed to evaluate the efficacy of phentolamine in prevention of CI-AKI in CKD and CCS patients undergoing percutaneous coronary catheterization for diagnostic angiography ± stenting.

**Methods:**

Participants with CKD and CCS planned for percutaneous coronary catheterization were included, while participants with normal kidney functions were excluded. A consecutive sample of 107 participants (mean age 58.62 ± 8.96 years, 64.5% males) was selected, underwent diagnostic coronary angiography or percutaneous coronary intervention, and received either conventional CI-AKI prevention strategy (group 1) or periprocedural phentolamine and conventional CI-AKI prevention strategy (group 2).

**Results:**

The percentages of study participants who had CI-AKI were 82.9% for group 1 and 17.1% for group 2, respectively. The incidence rate of CI-AKI was significantly lower in group 2 versus group 1 (*p* <  0.001). The urine output (ml/kg) and the urine output (ml/hour) within 72 hours post procedure was significantly higher in group 2 versus group 1 (t(105) = − 0.69, *p* <  0.001, t(105) = − 52.46, *p* < 0.001, respectively), the peak change in serum creatinine and the percentage of change relative to the baseline serum creatinine at 72 hours post procedure was significantly lower in group 2 versus group 1 (t(102) = 0.2, p 0.018, t(102) = 23.54, *p* < 0.001, respectively), and the incidence rate of major adverse cardiac and cerebrovascular events within 90 days post procedure was significantly lower in group 2 versus group 1 (t(102) = 1.168, *P* < 0.001), respectively. There was a statistically significant association of periprocedural phentolamine infusion with prevention of CI-AKI (OR = 0.041, 95% CI 0.0149–0.1128, *P* < 0.0001).

**Conclusion:**

Our study highlights the potential role of phentolamine for protection of the kidney in CKD patients planned for coronary catheterization.

**Trial registration:**

Pan African Clinical Trial Registry Number: PACTR202209493847741.

Date of Trial Registration: 22/09/2022.

## Introduction

Contrast induced acute kidney injury (CI-AKI) is the leading cause of hospital-acquired acute kidney injury (AKI) with a reported incidence rate of 1–6% in the general population [1]. More than half of the cases are reported in patients undergoing percutaneous coronary catheterization for diagnostic angiography ± stenting. A large, retrospective case-control study at Mayo clinic showed an overall 3.3% incidence rate of CI-AKI in patients who underwent primary percutaneous coronary intervention (PCI) [2]. Risk factors for CI-AKI include chronic kidney disease (CKD), diabetes mellitus (DM), age, hypertension, peripheral vascular disease (PVD), congestive heart failure (CHF), shock with systolic blood pressure < 100 mmHg, intra-aortic balloon pump, anemia with hemoglobin < 11 mg/dl, serum creatinine > 1.5 mg/dl, and contrast media (CM) volume > 260 ml [3, 4]. In 1992, Hall et al. showed that CI-AKI is 30 times higher in patients with baseline serum creatinine ≥2.0 mg/dl versus patients with baseline serum creatinine ≤1.2 mg/dl [5]. CI-AKI in CKD patients undergoing percutaneous coronary catheterization for diagnostic angiography ± stenting is associated with an overall mortality rate of 7–31%, dialysis treatment rate of less than 1%, and permanent dialysis treatment rate of less than 0.13% [6]. In 2015, Takahide Nawa et al. showed a significant lower incidence rate of CI-AKI in the study participants who received periprocedural hydration and the vasodilator agent nicorandil (2.0%) versus the study participants who received periprocedural hydration only (10.7%) (*P* < 0.02) and a statistically significant lower odds of developing CI-AKI with periprocedural hydration and the vasodilator agent nicorandil versus periprocedural hydration only (OR: 0.173, 95% CI 0.037–0.812, *P* = 0.026) [7]. Renal denervation and subsequent reduction of renal sympathetic activity decreased the incidence of microalbuminuria and macroalbuminuria by 10 and 23%, respectively, after 3 and 6 months without adversely affecting glomerular filtration rate in patients with resistant hypertension (*P* = 0.001) [8]. Phentolamine is a non-selective presynaptic alpha adrenergic receptor competitive blocker and a vasodilator drug used in treatment of emergency hypertension. We wanted to evaluate the efficacy of phentolamine in prevention of CI-AKI in patients with CKD and chronic coronary syndrome (CCS) undergoing diagnostic coronary angiography or PCI.

## Methods

### Study design

Our study was a 3-year prospective, open-labeled, randomized, controlled pilot study conducted at a single critical care unit (CCU) in a tertiary care hospital. This study was performed in accordance with the Egyptian National Commission for Bioethics (National Commission for UNESCO) statement on ethical conduct in human research, study procedures were carried out following the Code of Ethics of the World Medical Association (Declaration of Helsinki), study design and protocol were reviewed and approved by the human ethics committee of Cairo university, study was registered and issued a trial registration number (I-111015), study participants signed written informed consents, study data was anonymized, and the privacy rights of the study participants were observed diligently.

### Study participants

Study participants were CKD and CCS patients, candidate for diagnostic coronary angiography or PCI, and referred to the CCU. The study participants were subjected to history taking and data collection for age, gender, hypertension, DM, dyslipidemia, smoking, cerebrovascular disease, PVD, acute coronary syndrome (ACS), prior coronary angiography, and prior revascularization procedure. In addition, study participants were subjected to comprehensive clinical examination including measurement of vital signs on admission (systolic and diastolic blood pressure, heart rate, respiratory rate, and body temperature), mean arterial blood pressure, body weight and height, admission and discharge twelve-lead electrocardiograms (ECGs), transthoracic echocardiography (TTE), complete blood count, fasting blood glucose, blood urea, serum creatinine, total cholesterol, high density lipoprotein cholesterol, low density lipoprotein cholesterol, triglycerides, creatinine kinase (CK), creatine kinase-MB (CK-MB), and troponin. Normal values for ECG waves and intervals were referenced to the American College of Cardiology/American Heart Association Task Force on Assessment of Diagnostic and Therapeutic Cardiovascular Procedures (Committee on Electrocardiography) report [9]. Data documented with ECGs included arrhythmias, new bundle branch block, ST segment elevation, ST segment depression, changes of T wave, or no significant changes. Data documented with TTE included left ventricular ejection fraction (LVEF). The preprocedural CKD was categorized based on the estimated glomerular filtration rate (eGFR) using the simplified Modification of Diet in Renal Disease (MDRD) formula into mild CKD (60–89 ml/min/1.73 m^2^), moderate CKD (30–59 ml/min/1.73 m^2^), and severe CKD (15–29 ml/min/1.73 m^2^), respectively [10]. Screened participants were enrolled if they had CKD, CCS after previous episode of ACS including ST segment elevation myocardial infarction (STEMI), non-ST segment elevation myocardial infarction (NSTEMI), or unstable angina (UA), and planned for diagnostic coronary angiography or PCI. Screened participants with normal kidney functions, single functioning kidney, end stage renal disease (ESRD) on regular dialysis, history of kidney transplant, AKI triggered by cocaine, surgery, sepsis, trauma, or cardiogenic shock, pulmonary edema, acute heart failure with LVEF < 30%, systolic blood pressure < 80 mmHg, bronchial asthma, multiple myeloma, pregnancy, allergy to phentolamine or CM, or received barbiturates, antipsychotic agents, phosphodiestrase-5 inhibitors, CM within 7 days of study entry, or α-adrenoreceptor blocker at the time of admission were excluded from the study. Staging of CI-AKI was as per the Risk, Injury, Failure, Loss, and End-stage Kidney (RIFLE) criteria (Table 1). According to the Kidney Disease Improving Global Outcomes (KDIGO) work group, staging of CI-AKI shouldn’t be different from staging of AKI as per RIFLE criteria or the Acute Kidney Injury Network (AKIN) criteria [11].

**Table 1:** Staging of acute kidney injury

Stage of Acute Kidney Injury	Serum Creatinine	Urine Output
Stage 1	1.5–1.9 times baseline Or > 0.3 mg/dl (≥ 26.5 μmol/l) increase	< 0.5 ml/kg/hr. for 6–12 hrs
Stage 2	2.0–2.9 times baseline	< 0.5 ml/kg/hr. for ≥12 hrs
Stage 3	3.0 times baseline Or Increase in serum creatinine to ≥4.0 mg/dl (≥ 353.6 μmol/l) Or Initiation of Renal Replacement Therapy Or In patients, < 18 years, decrease in eGFR to < 35 ml/min/1.73 m^2^	< 0.3 ml/kg/hr. for ≥24 hrs Or Anuria for ≥12 hrs

### Study procedures

**Fig. 1:**
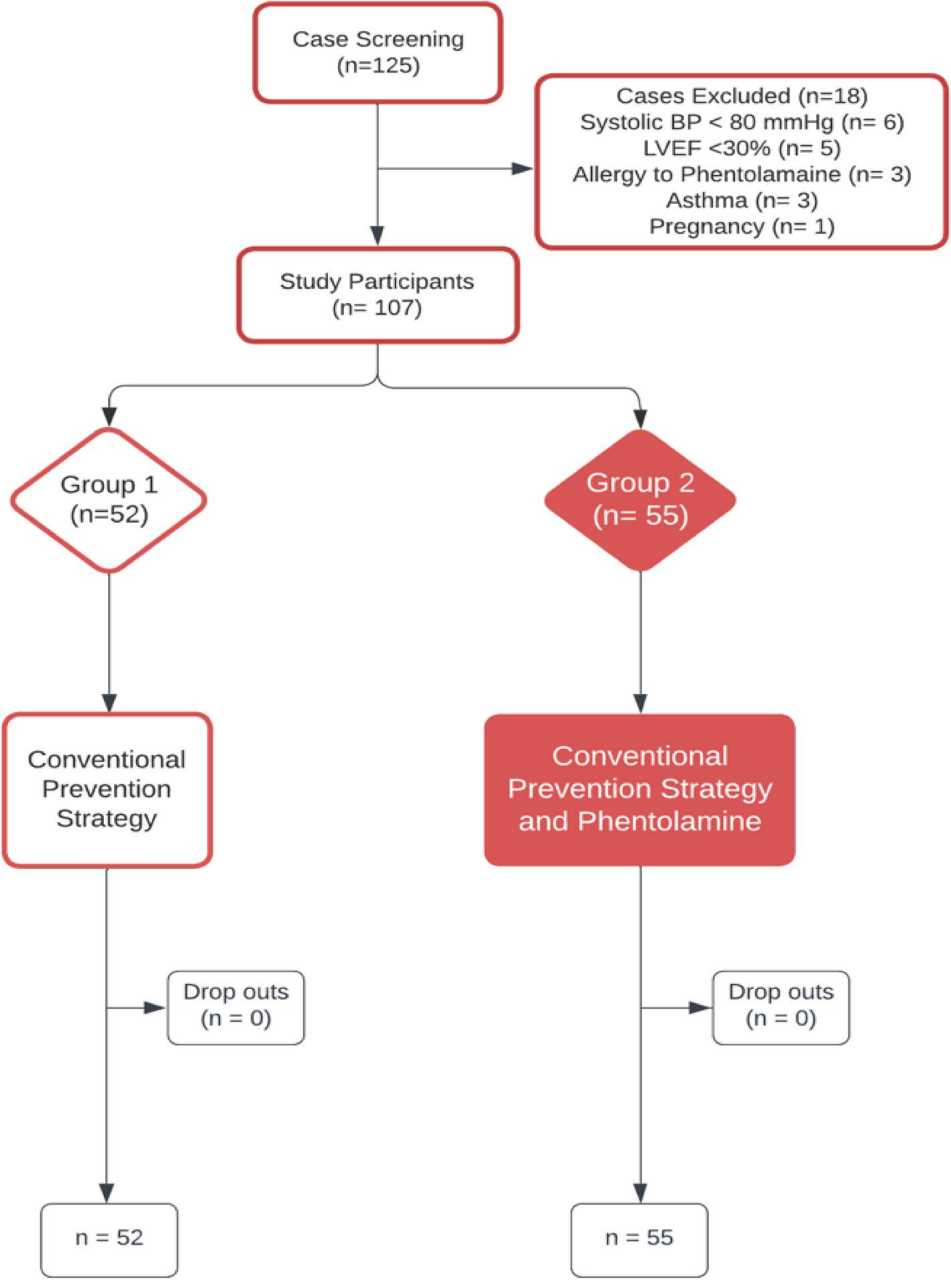
Case Screening and Processing Flow Chart showing case screening and processing

One hundred and seven eligible participants were randomly, consecutively assigned with an equal 1:1 allocation ratio into an open-labeled unblinded fashion. The enrolled study participants were randomized into 2 groups of 52 participants for conventional CI-AKI prevention strategy with normal saline infusion at a rate of 1–1.5 ml/kg/hr. to be started 12 hours before coronary angiography or PCI and continued for 24 hours after CM exposure in addition to 2 doses of N-acetylcysteine 600–1200 mg per oral the day before admission and 2 doses on the day of the procedure (Group 1) versus 55 age and gender matched participants for phentolamine 1–5 mg intravenous (IV) bolus followed by continuous IV infusion at a rate of 0.5–20 μgm/kg/min to be started 1 hour before coronary angiography or PCI and continued for 4–6 hours after CM exposure in addition to β-adrenoceptor blocker and conventional CI-AKI prevention strategy (Group 2) (Fig. 1).

### End points

The study evaluated the incidence of CI-AKI and association of periprocedural phentolamine with prevention of CI-AKI in CKD patients undergoing coronary catheterization. Secondary endpoints included change in urine output, serum creatinine and eGFR, and incidence of major adverse cardiac and cerebrovascular events (MACCE) in CKD patients undergoing coronary catheterization.

### Statistical analysis

Our study is a randomized controlled pilot study. The minimum anticipated observed effect size (correlation coefficient) couldn’t be estimated as there were no previously published or unpublished studies that assessed the efficacy of periprocedural phentolamine infusion in prevention of CI-AKI in CKD and CCS patients undergoing PCI for diagnostic angiography ± stenting. Accordingly, the minimum number of the study participants to be recruited (sample size) was based on feasibility. The assessment outcomes were coded, and the data was analyzed with the Statistical Package for the Social Sciences software (SPSS®) version 25. Quantitative (continuous) data was expressed as means and standard deviations, while qualitative (categorial) data was expressed as frequencies and percentages. Intention to Treat (ITT) principle was followed. Comparisons between parametrically distributed quantitative variables were done with the Independent two-tailed t-test, between non-parametrically distributed quantitative variables with Mann-Whitney test, and between qualitative variables with Chi-square test, respectively [12, 13]. The confidence interval was set to 95% and the margin of error accepted was set to 5%. Any comparison considered statistically significant was set at *P* < 0.05 or less and highly significant at *P* < 0.01.

## Results

### Study participants and procedures

We recruited 107 patients from one hospital in one country from October 2016 through November 2019. The 2 study groups were balanced with regards to the baseline characteristics and risk factors (Table 2). The key sociodemographic feature of the enrolled participants was male predominance (67.3% of group 1 and 61.8% of group 2 were males). Age was not significantly different between both groups (mean age was 58.5 ± 7.83 years for group 1 versus 58.73 ± 10.03 years for group 2, *P* > 0.914). Risk factors as hypertension, DM, smoking, ACS, prior coronary angiography, prior coronary artery bypass grafting, prior PCI, and history of CHF (variables believed to cause confounding) were equally distributed (matched) among both studied groups to adjust for confounding [14]. All enrolled participants completed the study and there were no withdrawals.

**Table 2:** Demographic data and patient characteristics

	**Group 1 (*N*** **= 52)**		**Group 2 (*N*** **= 55)**		***P*** **value**
	Mean ± SD		Mean ± SD		
Age (Years)	58.5 ± 7.83		58.73 ± 10.03		0.914
Baseline Serum Creatinine	1.65 ± 0.58		1.82 ± 0.39		0.847
Baseline eGFR	44.58 ± 10.06		38.41 ± 9.81		0.002
Ejection Fraction	54.17 ± 11.64		51.67 ± 10.46		0.233
	**Group 1 (*N*** **= 52)**		**Group 2 (*N*** **= 55)**		***P*** **value**
	N	%	N	%	
Male	17	32.7%	21	38.2%	0.553
Female	35	67.3%	34	61.8%	
Mild CKD	31	59.6%	15	27.3%	0.003
Moderate CKD	17	32.7%	30	54.5%	
Severe CKD	4	7.7%	10	18.2%	
Hypertension	42	80.8%	47	85.5%	0.517
Smoking	33	63.5%	31	56.4%	0.454
CABG History	2	3.8%	4	7.3%	0.679
CHF	31	59.6%	28	50.9%	0.365
STEMI	34	65.4%	44	80.0%	0.250
NSTEMI	12	23.1%	7	12.7%	
UA	6	11.5%	4	7.3%	
Old PCI once	12	23.1%	11	20.0%	0.703
Old PCI Twice	1	1.9%	3	5.5%	
Old CA once	24	46.2%%	27	49.1%	0.733
Old CA Twice	0	0.0%	1	1.8%	

*CHF* Congestive Heart Failure, *CA* Coronary Angiography, *CABG* Coronary Artery Bypass Graft, *PCI* Percutaneous coronary intervention, *NSTEMI* Non-ST segment myocardial infarction, *STEMI* ST segment elevation myocardial infarction, *UA* Unstable angina

### Incidence of contrast induced acute kidney injury

The incidence of CI-AKI in group 1 was 82.9% versus 17.1% in group 2. There was a significant difference in the incidence rate of CI-AKI between both groups (*P* < 0.001). There was no statistically significant difference among the incidence rates of CI-AKI as per the RIFLE criteria (38.3%), the KDIGO criteria (41.1%), and the AKIN criteria (42.1%) (*P* = 0.845) (Table 3).

**Table 3:** Incidence of Contrast Induced Acute Kidney Injury (CI-AKI) as per the Risk, Injury, Failure, Loss, and End-stage Kidney (RIFLE) Criteria, the Kidney Disease Improving Global Outcomes (KDIGO) Criteria, and the Acute Kidney Injury Network (AKIN) Criteria

		RIFLE		KDIGO		AKIN		*P* value
		Count	%	Count	%	Count	%	
CI-AKI	Yes	41	38.3%	45	42.1%	44	41.1%	0.845
	No	66	61.7%	62	57.9%	63	58.9%	

### Odds of contrast induced acute kidney injury

The odds of developing CI-AKI were > 95% lower among study participants who received periprocedural phentolamine and normal saline infusion than controls who received normal saline infusion only (OR = 0.041, 95% CI 0.0149–0.1128, *P* < 0.0001).

### Change in urine output

**Fig. 2:**
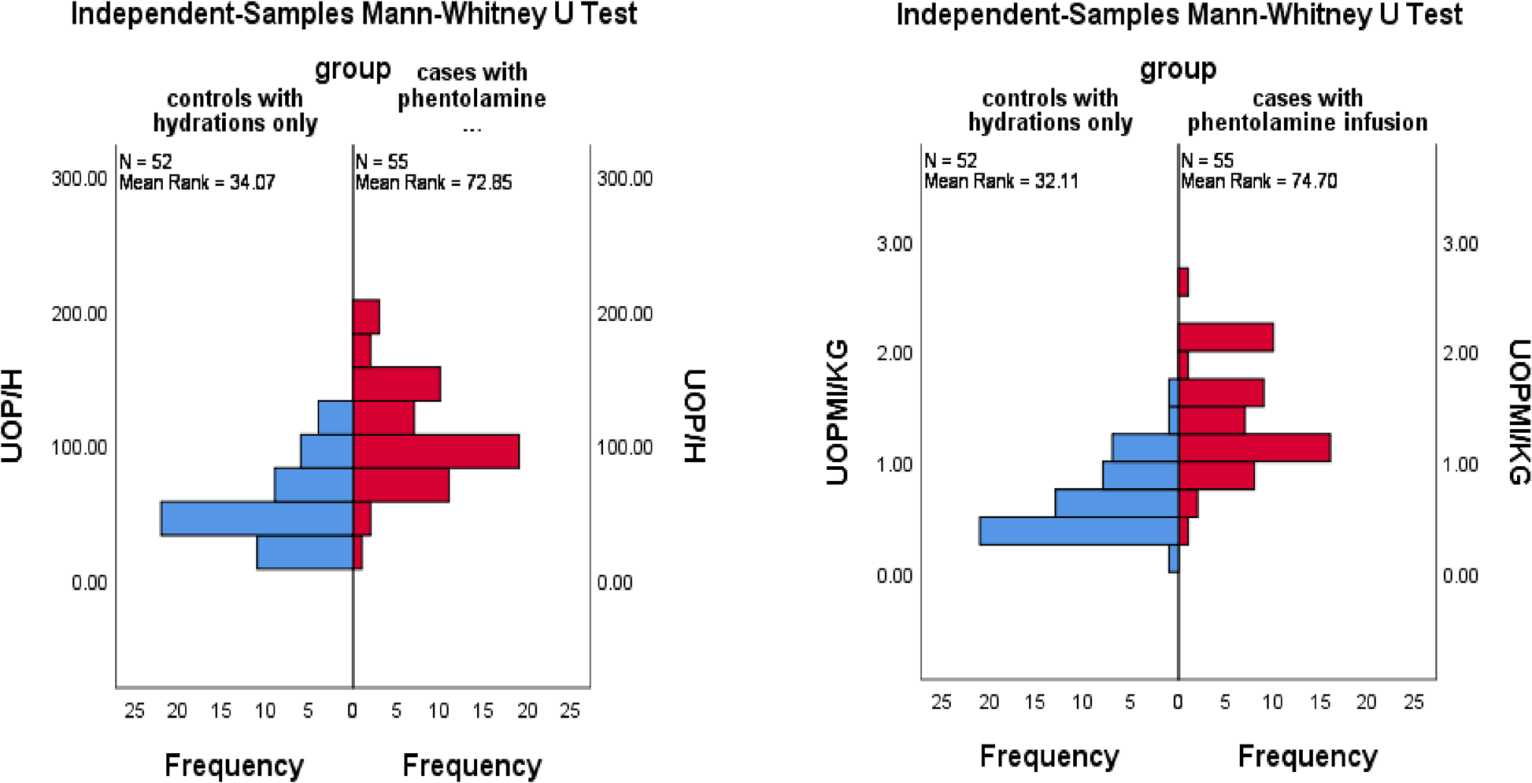
Histogram showing the change in urine output (ml/kg) in group 1 (mean urine output 0.64 ± 0.31 ml/kg) versus group 2 (mean urine output 1.33 ± 0.48 ml/kg) and the change in urine output (ml/hour) in group 1 (mean urine output 56.54 ± 28.14 ml/hr) versus group 2 (mean urine output 109.00 ± 39.20 ml/hr) within 72 hours post procedure

There were statistically significant differences between group 1 versus group 2 regarding the urine output (ml/kg) and urine output (ml/hour) within 72 hours post procedure (t(105) = − 0.69, *p* < 0.001 and t(105) = − 52.46, *p* < 0.001, respectively) (Fig. 2).

### Change in serum Creatinine and estimated glomerular filtration rate

**Fig. 3:**
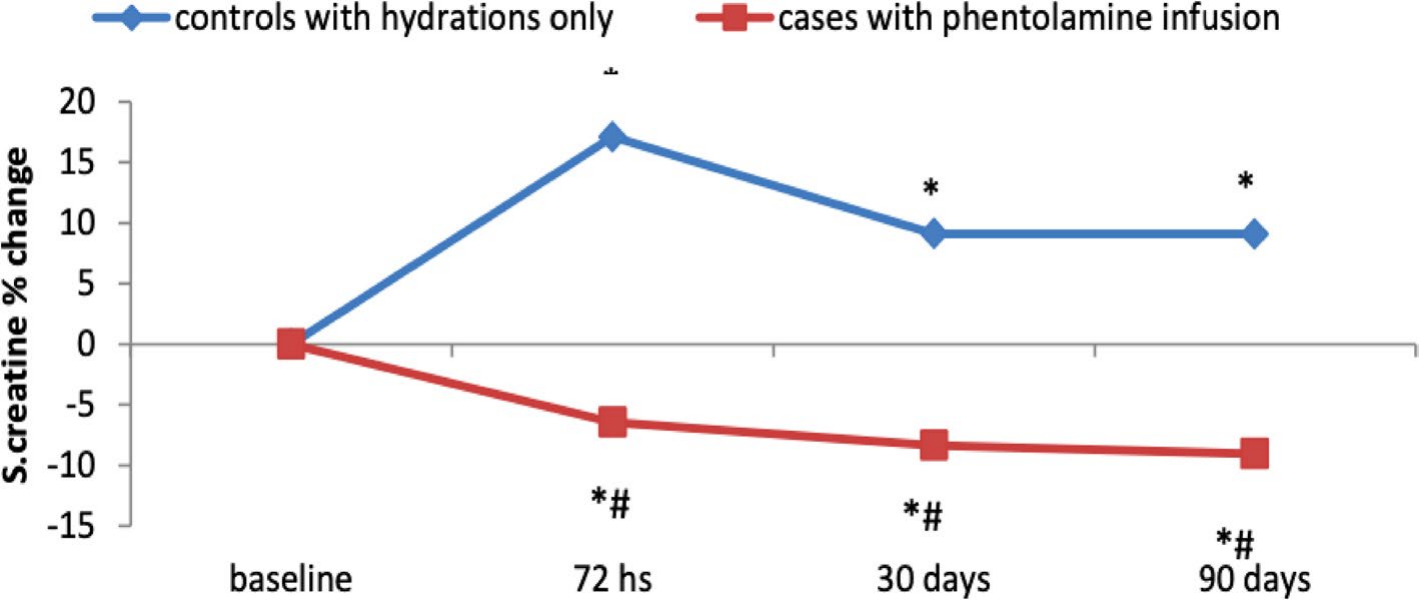
Line chart showing the percentage of change in serum creatinine relative to the baseline serum creatinine in group 1 at 72 hours post procedure (17.11 ± 15.93), 30 days post procedure (9.12 ± 23.06), and 90 days post procedure (9.10 ± 22.22) versus the percentage of change in serum creatinine relative to the baseline serum creatinine in group 2 at 72 hours post procedure (−6.43 ± 11.12), 30 days post procedure (−8.34 ± 15.83), and 90 days post procedure (−9.03 ± 15.59)

**Fig. 4:**
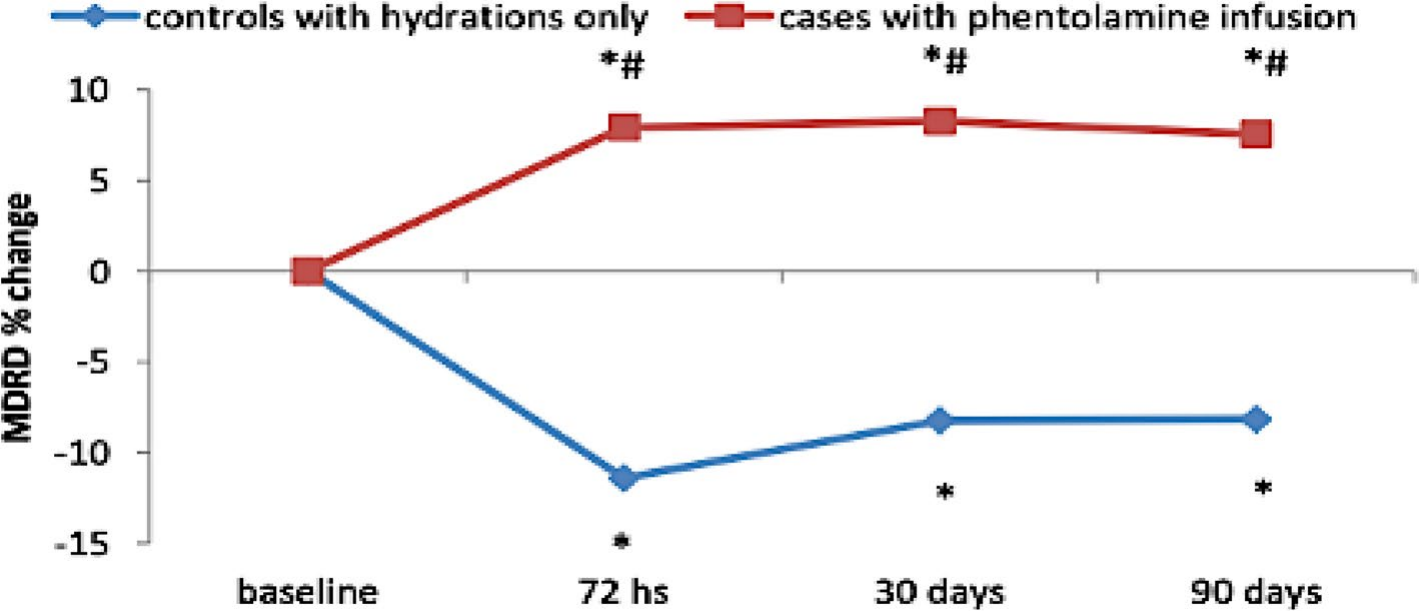
Line chart showing the percentage of change in eGFR relative to the baseline eGFR in group 1 at 72 hours post procedure (−11.41 ± 12.33), 30 days post procedure (− 8.23 ± 16.90), and 90 days post procedure (− 8.16 ± 17.07) versus the percentage of change in eGFR relative to the baseline eGFR in group 2 at 72 hours post procedure (7.93 ± 13.05), 30 days post procedure (8.28 ± 20.82), and 90 days post procedure (7.55 ± 24.93)

There were statistically significant differences between group 1 versus group 2 regarding the peak change in serum creatinine within 72 hours post procedure, percentage of change relative to the baseline serum creatinine at 72 hours post procedure, percentage of change relative to the baseline eGFR at 72 hours post procedure, percentage of change relative to the baseline serum creatinine at 30 days post procedure, percentage of change relative to the baseline eGFR at 30 days post procedure, percentage of change relative to the baseline serum creatinine at 90 days post procedure, and percentage of change relative to the baseline eGFR at 90 days post procedure (t(102) = 0.2, p 0.018, t(102) = 23.54, *p* < 0.001, t(102) = − 19.34, *p* < 0.001, t(102) = 16.53, *p* < 0.001, t(102) = − 16.51, *p* < 0.001, t(102) = 18.13, *p* < 0.001, and t(102) = − 15.71, *p* < 0.001, respectively) (Figs. 3 and 4).

### Incidence of major adverse cardiac and cerebrovascular events

The incidence of MACCE within 90 days post procedure in group 1 was 2.75 ± 0.926 versus 1.582 ± 0.994 in group 2. There was a significant difference in the incidence rate of MACCE between both groups (t(102) = 1.168, *P* < 0.001).

## Discussion

Percutaneous coronary catheterization for angiography and stenting has been extensively performed in coronary artery disease for revascularization. CI-AKI is a leading cause of hospital-acquired AKI with a reported annual incidence rate of 1–6% in the general population [1]. CI-AKI is associated with significant morbidity and mortality and can worsen cardiac, cerebrovascular, and patient reported outcomes. CKD, DM, age, and hypertension have been suggested as risk factors for CI-AKI [3, 4]. Periprocedural hydration with IV saline infusion is widely accepted as a cost effective CI-AKI prevention strategy. Using a vasodilator agent for prevention of CI-AKI was investigated by Takahide Nawa et al. in 2015 who showed a significant lower incidence rate of CI-AKI in the study participants who received periprocedural hydration and nicorandil (2.0%) versus the study participants who received periprocedural hydration only (10.7%) (*P* < 0.02) and a statistically significant lower odds of developing CI-AKI with periprocedural hydration and nicorandil versus periprocedural hydration only (OR: 0.173, 95% CI 0.037–0.812, *P* = 0.026) [7]. We have included oral N-acetylcysteine in our CCU conventional CI-AKI prevention strategy according to the KDIGO guideline 4.4.3 which advocates for using oral N-acetylcysteine with intravenous isotonic crystalloids in patients at increased risk of CI-AKI [11]. The effect of oral N-acetylcysteine on the incidence of CI-AKI is variable and the studies that concluded reduced incidence of CI-AKI with oral N-acetylcysteine showed heterogeneous results; most of the studies were of either high or modest quality. In one study, a protective dose-dependent effect was observed [15]. A recent systematic review and Bayesian network meta-analysis published in 2017 reported a statistically significant lower odds of developing CI-AKI with either periprocedural hydration, high-dose statin, and N-acetylcysteine versus periprocedural hydration only (OR = 0.31, 95% CI 0.14–0.60) or periprocedural hydration and high-dose statin versus periprocedural hydration only (OR = 0.37, 95% CI 0.19–0.64) in sensitivity analyses, meta-regression, and subgroup analyses, respectively [16]. In 2018, Weisbord et al. investigated the efficacy of IV saline, IV sodium bicarbonate, oral N-acetylcysteine, and oral placebo. CI-AKI occurred with comparable frequencies in all groups and the study concluded no differential benefit of IV saline, IV sodium bicarbonate, oral N-acetylcysteine, or oral placebo for the prevention of death, need for dialysis, persistent kidney impairment at 90 days post procedure, prevention of CI-AKI, or other secondary end points [17]. Another study by Xing K et al. showed that, despite the significant difference in the incidence rate of CI-AKI in the study participants who received periprocedural recombinant human B-type natriuretic peptide versus the study participants who received nitroglycerin (12.28% versus 28.81%, *P* < 0.05), there were non-significant differences between both groups in mortality and re-hospitalization within 3 months after PCI [18].

Our prospective study revealed a statistically significant difference in the incidence rate of CI-AKI as per the RIFLE criteria between the participants who received periprocedural hydration and phentolamine (17.1%) versus the participants who received periprocedural hydration only (82.9%) (*P* < 0.001), a statistically significant lower odds of developing CI-AKI with periprocedural hydration and phentolamine versus periprocedural hydration only (OR = 0.041, 95% CI 0.0149–0.1128, *P* < 0.0001), a statistically significant association between periprocedural phentolamine and prevention of CI-AKI, and a statistically significant difference in the incidence rate of MACCE between both groups within 90 days post procedure (t(102) = 1.168, *P* < 0.001).

## Strengths and limitations

Our study didn’t have missing data allowing robust per protocol analysis and the investigators who analyzed and reported the anonymous urine output, serum creatinine, and eGFR were blinded to the identity and clinical data of the study participants and hence minimizing observer bias. On the other hand, the study has limitations. It was a single centered study with a small sample size. Being a short prospective study with a lack of lengthy follow up didn’t allow us to investigate the chronological relationship between periprocedural phentolamine infusion and the long-term all-cause morbidity and mortality following cardiac catheterization.

## Conclusions and recommendations

CKD is a major risk factor for CI-AKI in CCS patients undergoing coronary catheterization. The significantly lower odds of developing CI-AKI among study participants who received periprocedural phentolamine highlights the potential role of periprocedural phentolamine infusion in improving postprocedural urine output and prevention of CI-AKI in CKD patients. Periprocedural phentolamine resulted in lower incidence of MACCE within 90 days post procedure and reduced short-term all-cause morbidity following cardiac catheterization. Large prospective studies with lengthy follow-up are warranted to assess the chronological relationship between periprocedural phentolamine infusion and long-term all-cause morbidity and mortality following cardiac catheterization.

## Data Availability

All data generated during this study are included in this published article and all datasets used and/or analysed are available from the corresponding author on reasonable request.
